# Change and Consistency of Self-Esteem in Early and Middle Adolescence in the Context of School Transition

**DOI:** 10.1007/s10964-019-01041-y

**Published:** 2019-05-27

**Authors:** Marta Białecka-Pikul, Małgorzata Stępień-Nycz, Iwona Sikorska, Ewa Topolewska-Siedzik, Jan Cieciuch

**Affiliations:** 1 Institute of Psychology, Jagiellonian University, Krakow, Poland; 2 Institute of Applied Psychology, Jagiellonian University, Krakow, Poland; 3 Institute of Psychology, Cardinal Stefan Wyszynski University, Warsaw, Poland; 4 University Research Priority Program Social Networks, University of Zurich, Zurich, Switzerland

**Keywords:** Self-esteem, Global self-esteem, Self-evaluations, Continuity, Stability, Developmental Change

## Abstract

Self-esteem is continuous and has stable characteristics, but it may also change, e.g., during transitions from one educational level to the next. In a prospective cross-sectional study over a year and a half, 250 Polish early adolescents (*N* = 109, 54 girls; mean age at T1 = 12.68 years, *SD* = 0.49) and middle adolescents (*N* = 141, 107 girls; mean age at T1 = 15.80, *SD* = 0.44) were tested three times using Harter’s Self-Perception Profile for Adolescents, assessing both global self-esteem and self-evaluation in eight domains. The change and consistency of self-esteem were analyzed, at both group and individual levels. At the group level, the following results were found: (1) continuity of self-esteem in five domains (scholastic competence, athletic competence, physical appearance, close friendship, and romantic appeal) and in global self-esteem and discontinuity in only three domains (social acceptance, job competence, and behavioral conduct); (2) significant inter-individual variation in the change not explained by age; and (3) higher self-esteem (in five domains) in early adolescents. At the individual level, the stability in most domains was weak, but was restored over the second year at the new school. The complexity of the developmental change and consistency in self-esteem in adolescence was highlighted, emphasizing the need for analyzing both group and individual change.

## Introduction

The main interest of this article is the evaluative aspect of self-concept, i.e., self-esteem (Rosenberg 1965; Harter 2012a). Self-esteem can be regarded as both a stable trait and an unstable state, as several events (both successes and failures) can impact momentary feelings of self-worth, particularly in specific domains of self-esteem (e.g., scholastic self-esteem; see: James 1890/1952; Crocker and Wolfe 2001). The adolescent period may be specifically dynamic with regard to self-esteem. The period is a time of dramatic developmental transitions in many domains of development, which influence changes in global self-esteem and its domains (Harter 2012b), as well as a time of changing social and educational contexts during school transitions (Harter 1990, 2006, 2012b).

The main aim of the present study was to describe the change and consistency of self-esteem during school transition. Following Bornstein et al. (2017), the term “consistency” is used to describe two main developmental qualities. The first is “continuity”, which may be defined as the lack of change at a group (i.e., mean level of the tested construct), which in this study is self-esteem. The second is “stability”, which may be defined as the lack of change at the individual (i.e., rank-order level), of the relevant construct, here self-esteem. Continuity and stability were tested with regard to global self-esteem and domain-specific self-evaluations. Also taken into account were the differences between early and middle adolescence with regard to the above-mentioned aspects of self-esteem. Therefore, from a more general point of view, the study aimed to compare early and middle adolescents’ self-esteem to discover differences between these two phases of development, and, at the same time, from a dynamic point of view, to explore change, continuity and stability in self-esteem during these two phases of adolescence.

### Global self-esteem and its domains in adolescence

Both Shavelson (Shavelson et al. 1976) and Harter (1987; 2012b) refer to “self-esteem” as a multidimensional, hierarchically-organized construct, with global self-esteem at the apex of the hierarchy. This multidimensionality is a core difference between these theories and Rosenberg’s (1965) concept of self-esteem, which represents a unidimensional approach. According to Harter (2012a), global self-esteem (she called it “self-worth”) refers to the general evaluation of how much one likes oneself and how much one is happy with oneself and the way one is as a human being. The specific domains of self-esteem in adolescence include scholastic competence, social acceptance, physical appearance, athletic competence, romantic appeal, close friendships, job competence and behavioral conduct (Harter 2012a, 2012b). It should be noted that, in this model, global self-esteem is not a simple resultant or mere sum of points in domain-specific dimensions of self-esteem, as specific dimensions can relate differently to global self-esteem (Harter 2012a).

Although early and middle adolescents’ self-esteem can be described using similar domains, several factors related to these two periods of development can influence the dynamics of self-esteem in different ways. First, early adolescence is a time of more rapid and dynamic changes associated with puberty that may influence one’s self-evaluation in domains related to physical appearance or athletic competence (Harter 2012b). The biological, pubertal changes influencing adolescents’ physical appearance and attractiveness may also influence their global self-esteem as physical appearance seems to be the most important domain for the formation of this general self-evaluation (Harter 2000). Second, social changes related to the period of early adolescence, including the change of social environment during the transition to junior high school (Wigfield et al. 1991) and the increasing role of social comparisons in the formation of self-esteem (Cole et al. 2001), may influence self-esteem in the domains related to social context. Third, the transition to junior high school and related changes in academic and behavioral requirements may influence these domains of self-esteem (Wigfield et al. 1991). Although the middle adolescence period is not free from changes in the physical, social and academic domains, the growing maturity of adolescents and decreasing maturity gap (see Moffitt 1993) may serve as protective factors that limit the fluctuation of self-esteem. In particular, neurodevelopmental processes during adolescence related to cognitive and affective development may influence self-esteem changes (Steinberg 2005; Dumontheil 2016).

### Change and consistency of self-esteem: a developmental perspective

The issue regarding self-esteem during adolescence that is still under debate is the change and consistency in the positivity/negativity of self-esteem. Empirical studies regarding this issue have produced contradictory results. Orth et al. (2012) reported an increase, i.e., discontinuity in global self-esteem, from age 16 to middle adulthood and then a decrease into old age. On the other hand, a meta-analysis of 59 longitudinal studies on global self-esteem during the lifespan (Huang 2010) revealed only a small increase in global self-esteem during childhood, late adolescence and early adulthood and generally supported the idea of its continuity during the lifespan. The effect sizes for the change in global self-esteem were similar to those observed in personality traits (Huang 2010; see also Orth and Robins 2014). Similarly, the meta-analysis of individual stability, i.e., rank-order stability of global self-esteem during the lifespan (Trzesniewski et al. 2003), indicated an increase in stability of global self-esteem from childhood to the first decade of adulthood and then a decrease from the second decade of adulthood to old age. Therefore, one can conclude that global self-esteem obtains its consistent level in early adulthood, both at the mean and individual levels. Moreover, both meta-analyses revealed no effect of gender, suggesting a similar pattern of change for males and females (Huang 2010; Trzesniewski et al. 2003).

When concentrating on adolescence, the results relating to continuity and change in self-esteem are more contradictory. Theoretically, dynamic developmental changes during the adolescent period can result in a decrease in self-evaluations at a group level, and the tantrums of adolescence can be a specifically sensitive period in this area (Harter 2012b). Some cross-sectional (Robins et al. 2002) and longitudinal (Baldwin and Hoffman 2002) studies on self-esteem confirm this hypothesis, reporting a drop in global self-esteem between childhood and adolescence and between early and middle adolescence (Eccles et al. 1993). However, in other studies, this decrease was observed only in some domains of self-esteem and not for global self-esteem (Shapka and Keating 2005; Kuzucu et al. 2014). In Kuzucu et al. (2014) longitudinal study of children between 9–16 years old, a significant—although only minor—decrease was found in the domains of physical appearance and behavioral conduct, accompanying an increase in the domains of athletic competence, social acceptance and academic competence. Global self-esteem remained continuous during this 7-year period of time. Some data also indicate continuity of self-esteem during the adolescence period, both regarding global self-esteem (Birkeland et al. 2012; Kuzucu et al. 2014) as well as specific dimensions of self-esteem (Young and Mroczek 2003). Finally, some studies have reported an increase in global self-esteem between early and middle adolescence (Moneta et al. 2001) and beyond (Erol and Orth 2011).

The discrepancies in these results regarding the continuity and change of self-esteem may stem from several reasons. One may be a differentiated conceptualization of self-esteem, with a focus on either global self-esteem, measured using different questionnaires, or on its specific dimensions (Kuzucu et al. 2014). The other may be tied to the different ages of participants as adolescents in the early and middle phase of this period face different developmental changes and challenges that may influence their self-esteem (Harter 2012b). Moreover, most studies included only a group level of analysis, studying changes in the mean level of self-esteem, whereas substantial individual differences regarding the trajectories of self-esteem have been observed (Kuzucu et al. 2014). For example, Harter and Whitesell (2003) reported no change in the mean value of global self-esteem in adolescents transitioning from high school to college. However, at the individual level, the pattern of results was much more differentiated, with a decrease of global self-esteem in 17% of adolescents, an increase in 23% and stability in 40% of the group. Therefore, a strong need also exists to explore individual stability and change in self-esteem in early and middle adolescence. This claim is also supported by results of studies using latent growth modeling, which indicate that individual differences exist in global self-esteem, in specific domains of self-esteem and also in change in self-esteem during adolescence (Young and Mroczek 2003; Birkeland et al. 2012). Finally, the measures used to assess global self-esteem may also be an important factor influencing the results. The meta-analysis by Huang (2010) revealed an important effect of using Harter’s Self-Perception Profile for Adolescence (SPPA; 2012a), which differs from both the Rosenberg Self-Esteem Scale (Rosenberg 1965) and the Coopersmith Self-Esteem Inventory (Coopersmith 1981). The differences may be due to a different conceptualization of the construct of global self-esteem (see Harter 2012a). It also should be noted that the observed patterns of change in self-esteem may depend on the time intervals taken into account, as some life events may create contexts for short-term changes in self-esteem (Chung et al. 2014).

### School transition as the context for self-esteem changes

The decrease in global self-esteem during childhood and adolescence may be related to the experience of stressful events (Baldwin and Hoffmann 2002), one of which may be school transitions (Wigfield et al. 1991; Eccles et al. 1993; Chung et al. 2014). School transitions can be a stressful event as it involves changes in academic demands and social contexts (Harter et al. 1992), involving a greater emphasis on evaluation and social comparison among students, stricter grading standards and a disruption of young adolescents’ social networks (Wigfield et al. 1991). Therefore, the domains of academic performance and social acceptance, as well as global self-esteem, may be mostly prone to change during this time. Seidman et al. (1994) reported a decline in both self-esteem and perceived positivity of peer context (e.g., perceived social support) during school transition in early adolescence among poor youth. However, Cantin and Boivin (2004) observed an increase in the self-evaluation of social acceptance during the transition from elementary school to junior high school, likely due to the intensification of supportive relationships with school friends. On the other hand, they observed a decrease in perceived scholastic competence, followed by a decrease in global self-esteem. As Cole et al. (2001) indicated, school transitions for many adolescents may be related to the discontinuity of self-esteem; however, the direction of change may differ depending on the developmental period as they observed a drop in perceived academic competence during middle school transition but an increase in this domain during transition into high school (Cole et al. 2001). Moreover, in early adolescence, a significant drop in continuity of self-perceived competence in most domains was observed, whereas in middle adolescence, self-evaluations in most domains increased and reached their plateau (Cole et al. 2001), suggesting that school transition, combined with developmental changes of early adolescence, may create a context that is particularly challenging for young people and their self-evaluation (see also Simmons et al. 1987). On the other hand, studies also reported a lack of decrease (or even an increase) in self-esteem and perceived competence during transition from elementary to junior high school (Hirsch and Rapkin 1987; Proctor and Choi 1994), indicating a need for further research.

## Current Study

The main aim of the present prospective cross-sectional study was to assess the continuity and stability of self-esteem in early and middle adolescence by focusing on global self-esteem and domain-specific self-evaluations. Moreover, it aimed to observe the short-term trajectory of self-esteem development just after the school transition for both age groups. The combined analyses of changes observed at the mean—or group—level and individual—or rank-order—level would fill an important gap in studies on self-esteem in adolescence. Moreover, analyzing the changes in both early and middle adolescence in the context of school transition may add an important contribution to understanding differences between younger and older adolescents, providing both a more detailed picture of the challenges they face, as well as implications for the support of their development.

Given the importance of school transitions, it should be specified that the Polish educational system has several transitions, and only one of them is obligatory: the transition from elementary school (which lasts for 6 years) to junior high school (3 years). After junior high school, several options are available: it is possible to end formal education or to transition to the next level, which may be high school (about 50% of adolescents choose this), technical college or vocational school. The current study included adolescents after the transition to junior high school and high school.

Taking into account that the adolescent period and its challenges (among them, school transition) may create fluctuations in self-esteem, a decrease in self-esteem over the tested period, i.e., in the context of school transition, was hypothesized (Hypothesis 1). As several studies have shown that after the initial decrease in self-esteem in early adolescence, a growth of self-esteem in later adolescence and adulthood is observed, it was expected that self-esteem in middle adolescence would be higher than in early adolescence, both regarding global self-esteem and specific domains (Hypothesis 2a) and that the decrease observed in self-esteem in early adolescence would be steeper than in middle adolescence (Hypothesis 2b). Taking into account the individual level of analyses, it was hypothesized (Hypothesis 3) that there is a stability of individual differences in self-esteem throughout adolescence over a year and a half, and this stability may be higher in middle than in early adolescence, as previous studies have revealed considerably high (increasing with age) rank-order stability of self-esteem.

## Method

### Participants

Participants included 250 Polish adolescents, assessed for the first time just after the transition into a new school (during the first month in the new school; see “Procedure” for details). The amount of missing data was 34.4% in the second wave (time 2, T2^1^) and 9.6% in the third wave (time 3, T3). The adolescents who provided data on each time point did not differ from the group that lacked some measurement points regarding gender (χ^2 ^= 0.30, *p* = 0.54) and affluence (*t* = 0.40, *p* = 0.69) but differed in age (*t* = 3.07, *p* < 0.01) as there were more middle adolescents (MA) who missed some measurement point, in comparison to early adolescents (EA): *n* = 61 versus *n* = 26, respectively. When looking separately at early and middle adolescence groups, no age differences existed between the groups with and without some missing data (EA: *t* = 0.12, *p* = 0.91; MA: *t* = −0.72, *p* = 0.47).

To check the pattern of missingness, Little’s (1988) Missing Completely at Random test (MCAR) was conducted. The results suggested that in the whole sample (χ^2^ = 22948, *df* = 18, *p>* 0.05), separately in the group of early adolescence (χ^2^ = 13.45, *df* = 18, *p>* 0.05) and middle adolescence (χ^2^ = 22.57, *df* = 18, *p>* 0.05), the data were missing completely at random. This enabled analyses to include data from all participants. As different analyses were used to test the hypotheses, missing data were estimated using the Expectation-Maximalization algorithm (EM; Klein and Moosbrugger 2000; Rubin and Thayer 1982) to implement the data exactly in all statistical procedures. Finally, the younger group consisted of 109 13-year-old students from junior high school (54 girls and 55 boys; mean age at T1 *M* = 12.68 years, *SD=* 0.49; at T2 *M=* 13.42, *SD* = 0.48; at T3 *M=* 13.73, *SD* = 0.44), while the older group consisted of 141 16-year-old students from high school (107 girls and 34 boys; mean age at T1 *M* = 15.80, *SD=* 0.44; at T2 *M* = 16.47, *SD* = 0.44; at T3 *M* = 16.74, *SD* = 0.44).

Participants were recruited from five state schools, mainly in an urban area of Kraków, Poland. The first language for 98% of participants was Polish, and 2% came from a mixed or bilingual background. Such homogeneity is typical in Poland. Most of the participants were from middle socioeconomic families: 26% were classified as high affluence, 56% as middle affluence and 18% as low affluence, as measured by the Family Affluence Scale III (FAS III; Mazur 2013; Torsheim et al. 2016). None of participants had problems with general cognitive skills (as measured using a standardized instrument for assessing language ability: General Test of Word Comprehension–Standard Version; Matczak et al. 2012).

### Measures

As the multidimensional view of self-concept was adopted in this study, following Harter’s definition, the Self-Perception Profile for Adolescents (SPPA) was used to measure self-esteem. In accordance with Harter’s definition of self-concept, the SPPA is an instrument designed to measure an adolescent’s overall self-esteem and feelings of competence in eight specific domains, namely: scholastic competence, social acceptance, athletic competence, physical appearance, behavioral conduct, romantic appeal, job competence and close friendship (see Harter 1988; 2012a). The SPPA also provides a global self-esteem score, which shows the extent to which the adolescent likes himself/herself as a person and is happy with the way he/she is. Therefore, the SPPA consists of nine subscales, with five items each. In other words, both the original American SPPA and the Polish SPPA are comprised of a total of 45 items. Table 1 presents sample items of each of the subscales of the SSPA (Harter 2012a).^2^

**Table 1:** Examples of the items for each subscale of the SPPA

Domain of self-esteem	Example of the item
Global self-esteem	Some teenagers are happy with themselves most of the time BUT Other teenagers are often not happy with themselves.
Scholastic competence	Some teenagers feel like they are just as smart as others their age BUT Other teenagers aren’t so sure and wonder if they are as smart.
Social acceptance	Some teenagers know how to make classmates like them BUT Other teenagers don’t know how to make classmates like them.
Athletic competence	Some teenagers do very well at all kinds of sports BUT Other teenagers don’t feel that they are very good when it comes to sports.
Physical appearance	Some teenagers think that they are good looking BUT Other teenagers think that they are not very good looking.
Job competence	Some teenagers feel that they are ready to do well at a part-time job BUT Other teenagers feel that they are not quite ready to handle a part-time job.
Romantic appeal	Some teenagers feel that if they are romantically interested in someone, that person will like them back BUT Other teenagers worry that when they like someone romantically, that person won’t like them back.
Behavioral conduct	Some teenagers usually do the right thing BUT Other teenagers often don’t do what they know is right.
Close friendship	Some teenagers are able to make really close friends BUT Other teenagers find it hard to make really close friends.

Importantly, the original American version of the SPPA was translated into the Polish language following gold standard recommendations (Brislin 1970). The translation was carried out in four steps: (1) two independent translators provided the first translations, (2) an expert panel consisting of the translators and psychologists discussed and resolved the discrepancies, (3) the Polish SPPA was translated back into American English by the independent translator, and (4) the back-translation was accepted by the author of the original questionnaire after making some corrections proposed by the author.

The response format of the SPPA—rating both global and specific domains of self-esteem—includes both positively and negatively worded phrases, including, for example, “some kids have a lot of friends”, but “others don’t have many friends”. The adolescent assesses his/her similarity to one of these two opposite statements and chooses not only which is “true for me” but also how much it is “true” (from “really true” to “sort of true”). Therefore, it uses a 4-point Likert scale, and the results are expressed as the means of each subscale of the SPPA.

In the present study, the average Cronbach’s alpha values ranged from 0.52 to 0.92 for each specific domain and thus reached acceptable (and similar to the original scale; Harter 2012a) levels for most domains (see Table 2 in the “Results” section).

**Table 2:** Descriptive statistics of self-esteem ratings in early and middle adolescence

Time of assessment	SPPA	Early adolescence			Middle adolescence		
		α	*M (SD)*	Min–Max	α	*M (SD)*	Min–Max
1	Scholastic competence	0.68	2.79 (0.58)	1.20–4.00	0.74	2.57 (0.66)	1.00–4.00
	Social acceptance	0.69	3.17 (0.58)	1.80–4.00	0.77	2.89 (0.69)	1.00–4.00
	Athletic competence	0.77	2.75 (0.73)	1.00–4.00	0.87	2.40 (0.80)	1.00–4.00
	Physical appearance	0.83	2.58 (0.74)	1.00–4.00	0.90	2.11 (0.82)	1.00–4.00
	Job competence	0.66	2.48 (0.58)	1.20–3.80	0.77	2.62 (0.64)	1.40–4.00
	Romantic appeal	0.56	2.65 (0.55)	1.40–4.00	0.66	2.60 (0.61)	1.40–4.00
	Behavioral conduct	0.64	2.47 (0.58)	1.00–3.40	0.60	2.36 (0.56)	1.20–3.60
	Close friendship	0.79	3.11 (0.69)	1.00–4.00	0.85	3.26 (0.75)	1.00–1.00
	Global self-esteem	0.80	2.99 (0.64)	1.00–4.00	0.86	2.55 (0.78)	1.00–4.00
2	Scholastic competence	0.74	2.69 (0.60)	1.20–4.00	0.53	2.62 (0.49)	1.50–4.00
	Social acceptance	0.73	2.91 (0.61)	1.00–4.00	0.81	2.81 (0.63)	1.00–4.00
	Athletic competence	0.79	2.70 (0.70)	1.00–4.00	0.88	2.44 (0.74)	1.00–4.00
	Physical appearance	0.85	2.46 (0.76)	1.00–4.00	0.88	2.33 (0.75)	1.00–4.00
	Job competence	0.68	2.56 (0.59)	1.20–4.00	0.79	2.64 (0.59)	1.20–4.00
	Romantic appeal	0.52	2.52 (0.49)	1.00–4.00	0.65	2.54 (0.51)	1.40–4.00
	Behavioral conduct	0.64	2.65 (0.53)	1.00–4.00	0.70	2.62 (0.47)	1.60–4.00
	Close friendship	0.77	3.00 (0.67)	1.00– 3.40	0.92	3.06 (0.74)	1.00–4.00
	Global self-esteem	0.85	2.72 (0.75)	1.00–4.00	0.86	2.61 (0.67)	1.00–4.00
3	Scholastic competence	0.68	2.68 (0.58)	1.00–4.00	0.67	2.59 (0.56)	1.20–3.60
	Social acceptance	0.75	2.92 (0.62)	1.40–4.00	0.77	2.81 (0.64)	1.00–4.00
	Athletic competence	0.79	2.72 (0.67)	1.20–4.00	0.86	2.41 (0.77)	1.00–4.00
	Physical appearance	0.78	2.49 (0.69)	1.00–4.00	0.86	2.33 (0.78)	1.00–4.00
	Job competence	0.70	2.57 (0.58)	1.40–4.00	0.74	2.71 (0.63)	1.00–4.00
	Romantic appeal	0.59	2.60 (0.51)	1.20–4.00	0.56	2.64 (0.55)	1.00–4.00
	Behavioral conduct	0.71	2.71 (0.60)	1.00–4.00	0.74	2.59 (0.58)	1.40–4.00
	Close friendship	0.77	3.07 (0.68)	1.00–4.00	0.86	3.12 (0.74)	1.00–4.00
	Global self-esteem	0.84	2.75 (0.70)	1.00–4.00	0.83	2.64 (0.71)	1.00–4.00

### Procedure

The presented results are part of a larger longitudinal study (see: “Acknowledgements”). The data collection consisted of group and individual techniques used at three time points: T1 (September 2014, the first month in a new school—junior high school in the younger group and high school in the older group), followed by T2 within the same schools and classes almost a year later (June 2015) and T3 half a year later (January 2016). The present study focused on data gleaned from each time during group sessions. The SPPA and two other questionnaires not relevant to the results presented here were always completed during the first group session at each measurement point. General information about students (e.g., gender, age) and their family wealth were collected during the second group sessions at T1. During this session, a language measure to control for general cognitive skills was also administered.

### Analytical strategy

In order to answer the research questions, two types of change and consistency in self-esteem were investigated: mean-level (continuity) and rank-order (stability). Furthermore, potential effects of the developmental period (early and middle adolescence) in moderating the patterns of continuity/stability and change were investigated, and two aspects of self-esteem were taken into account: global self-esteem and the ratings of domain-specific self-evaluations. In the study of self-esteem, mean-level change refers to the average change in self-esteem that a group ascribes to each value. Rank-order stability refers to the degree to which the relative ordering of individuals on self-esteem remains the same over time.

Latent Growth Curve (LGC) modeling was conducted to analyze the change in self-esteem at the group level, and in next step, the conditional LGC was conducted as an alternative model, introducing the age group into each model as a covariate to assess differences between age groups in its initial level and change. Then, to assess the rank-order stability of self-esteem, correlational analysis was conducted, and autoregressive models in structural equation modeling (SEM) were analyzed. All analyses were conducted using SPSS and Mplus (Muthén and Muthén 1998–2012) software. All conducted analyses and obtained results are presented below.

## Results

### Descriptive statistics

Descriptive statistics for the level of self-esteem in all three waves of the study were calculated (see Table 2).

### Mean-level change in self-esteem in early and middle adolescence

Latent Growth Curve modeling (LGC) is a group of statistical analyses used to assess construct-level changes over time, in which two kinds of growth parameters are computed. First is the *intercept*, which corresponds to the initial level of the construct; second are *slopes*, which are indicators of the change rate, i.e., linear or curvilinear. The growth factors provide information about general changes in the construct level (by significance of its mean) and inter-individual differences in parameter value (by significance of its variance). The LGC models were built to test the linear changes in global and specific self-esteem (Hypothesis 1), separately for each variable. The goodness of model fit was assessed using the following indicators, with expected values as CFI ≥ 0.90, RMSEA < 0.05, and SRMR < 0.05 (Hu and Bentler 1999). The results of LGC are presented in Table 3.

**Table 3:** The model fit indicators for LGC models

Variable	χ^2^*(df)*	CFI	RMSEA	SRMR	Intercept		Linear slope	
					*M*	*V*	*M*	*V*
Scholastic competence	0.177 (1)	1.00	0.000 [0.000; 0.126]	0.006	2.67***	0.13***	−0.03	0.02
Social acceptance	6.500 (1)	0.981	0.148 [0.057; 0.265]	0.027	2.98***	0.27***	−0.11**	0.12**
Athletic competence	0.059 (1)	1.00	0.000 [0.000; 0.105]	0.002	2.56***	0.40***	−0.01	0.11*
Physical appearance	0.568 (1)	1.00	0.000 [0.000; 0.151]	0.009	2.33***	0.37***	0.06	0.14*
Job competence	0.065 (1)	1.00	0.000 [0.000; 0.107]	0.003	2.56***	0.15***	0.07*	0.06
Close friendship	11.01 (1)	0.950	0.200 [0.106; 0.314]	0.036	3.17***	0.35***	−0.07	0.17**
Behavioral conduct	26.67 (3)	0.868	0.178 [0.120; 0.242]	0.168	2.46***	0.15***	0.17***	0.000*m*
Romantic appeal	10.12 (1)	0.927	0.191 [0.097; 0.305]	0.043	2.58***	0.15***	0.01	0.07
Global self-esteem	2.721 (1)	0.993	0.083 [0.000; 0.208]	0.020	2.72***	0.28***	−0.02	0.12*

*M* mean, *V* variance, *m* because of a non-positive covariance matrix growth factor’s variance was fixed to 0

*p< 0.05; **p < 0.01; ***p < 0.001

In the case of the majority of variables, all indicators suggested a good fit to the data. The exceptions included: social acceptance, where RMSEA was slightly above the expected value; global self-esteem; close friendship; romantic appeal, with RMSEA outside the good-fit ranges; and behavioral conduct, with RMSEA and SRMR too high and CFI below the cut-off point. On the basis of the Kenny et al. 2015, RMSEA values could indicate poor model fit in the models with small degrees of freedom, particularly for small sample sizes, which is the case in the present study. In line with this finding, RMSEA values in conducted analyses should be interpreted with caution and other indicators, such as CFI and SRMS, should be taken into account first of all. The first variables had at least one indicator suggesting good model fit. Only in the case of behavioral conduct did the results indicate weak model fit. Additionally, in the case of the variable problems, a positive covariance matrix was revealed. In conclusion, the results of behavioral conduct should be interpreted with carefulness.

With regard to Hypothesis 1, it can be concluded that social acceptance decreases, while job competence and, eventually, behavioral conduct increase with time in the tested period of one and a half years after the school transition. Significant variance of intercepts indicate that participants differ in the initial level of self-esteem in the case of both global self-esteem and all specific self-evaluations. Additionally, significant slope variance suggests that the change rate of social acceptance, athletic competence, physical appearance, close friendship and global self-esteem is inter-individually differentiated.

To test age differences in the initial level and the change rate of self-esteem (Hypotheses 2a and 2b), a set of conditional LGCs, with the age group factor as a covariate, was conducted. Obtained results are presented in Table 4.

**Table 4:** Standardized results from conditional LGC with age group as a covariate

Variable	Intercept	Linear slope
Scholastic competence	−0.477*	0.495
Social acceptance	−0.470**	0.326
Athletic competence	−0.491**	0.017
Physical appearance	−0.623***	0.485*
Job competence	0.315	0.001
Close friendship	0.230	−0.172
Behavioral conduct	−0.257	–
Romantic appeal	−0.111	0.283
Global self-esteem	−0.690***	0.590**

Standardized coefficients under the STDY standardization type. Early adolescents are coded as 1 and older as 2

*p < 0.05; **p< 0.01; ***p < 0.001

Age differences at the beginning of the new school (T1, i.e., the first measurement point) occurred in scholastic competence, social acceptance, athletic competence, physical appearance and global self-esteem. In all cases, the level of variables was higher in the younger group. No differences were found in the rate of change between the age groups, except for physical appearance and global self-esteem, although in the entire group, these variables obtained no significant course of change.

### The rank-order stability of self-esteem in the context of school transition

To verify the third hypothesis, i.e., to assess rank-order stability of self-esteem over three measurement points during the first year at the new school, SEM analysis was conducted. The autoregressive models were calculated for each domain of self-esteem, separately for early and middle adolescence. The self-esteem at T2 was regressed on its value at T1, and self-esteem at T3 was regressed on both T1 and T2 measurements. When used, the model could be seen as an example of a fully recursive Marcov chain model with time-based effects (McAdle and Nesselroade 2014). The model was fully constrained, and no fit indicators were calculated. The results of this analysis are presented in Table 5.

**Table 5:** Standardized coefficients for autoregressive models of self-esteem at three time points (T1, T2, T3)

Domain of self-esteem	Age group	Standardized regression coefficients			*R* ^*2*^	
		T2 on T1	T3 on T2	T3 on T1	T2	T3
Global self-esteem	EA	0.37***	0.59***	0.17*	0.14*	0.45***
	MA	0.60***	0.60***	0.12*	0.36***	0.61***
Scholastic competence	EA	0.47***	0.61***	0.16*	0.22**	0.49***
	MA	0.45***	0.64***	0.22**	0.21**	0.58***
Social acceptance	EA	0.53***	0.67***	0.13	0.29***	0.56***
	MA	0.60***	0.65***	0.11	0.36***	0.52***
Athletic competence	EA	0.63***	0.63***	0.19**	0.40***	0.58***
	MA	0.67***	0.83***	0.09	0.45***	0.80***
Physical appearance	EA	0.49***	0.62***	0.13	0.24**	0.49***
	MA	0.61***	0.75***	0.12*	0.37***	0.68***
Job competence	EA	0.49***	0.50***	0.21*	0.24**	0.40***
	MA	0.41***	0.67***	0.17**	0.17**	0.58***
Romantic appeal	EA	0.44***	0.41***	−0.06	0.19**	0.15*
	MA	0.41***	0.50***	0.24**	0.17**	0.41***
Behavioral conduct	EA	0.36***	0.50***	0.17*	0.13*	0.35***
	MA	0.39***	0.60***	0.23***	0.16**	0.52***
Close friendship	EA	0.60***	0.55***	0.10	0.36***	0.37***
	MA	0.50***	0.58***	0.14	0.25***	0.43***

*EA* early adolescence, *MA* middle adolescence

*p< 0.05; **p < 0.01; ***p < 0.001

The results presented in Table 5 suggest the stability of self-esteem in all domains as all regression coefficients are statistically significant. One should note, however, that for some domains (especially global self-esteem in early adolescence and behavioral conduct in early adolescence), the size effects were very small (*R*^*2*^ = 0.14 and 0.13, respectively), suggesting some lack of stability in these domains between T1 and T2. Additionally, for other domains of self-esteem, in both early and middle adolescence, the size effects were small to medium, and only in some domains, the percent of explained variance exceeded 50. This is particularly true for T2 regressed on T1, suggesting some lack of stability in self-esteem just after school transition. One can also note that, generally, size effects in middle adolescence were larger than those in early adolescence. Particularly stable domains in this age group included athletic competence and physical appearance as their level during the second and third measurement was strongly determined by their previous levels.

## Discussion

Previous studies on the development of self-esteem have concentrated mainly on the description of the change observed at mean (or group) level in global self-esteem in the context of school transitions. In this study, Harter’s Self-Perception Profile for Adolescents was used and thus both global self-esteem and self-evaluations in eight domains were assessed. Moreover, these were assessed three times over the period of a year and a half in two age-groups: early and middle adolescents (i.e., 13- and 16-year-olds). Moreover, both the change and the consistency of global self-esteem and domains’ self-evaluations were analyzed, taking into account both group (mean) and individual level. This seems particularly important, as the pattern of change or consistency may differ depending on the level of analysis. Additionally, a comparison was conducted between patterns of development observed during early and middle adolescence.

On the whole group level, a significant change following school transition was observed in three domains of self-esteem: increase in behavioral conduct and job competence and decrease in social acceptance. The remaining five domains of self-esteem, as well as global self-esteem, remained stable during the 18 months following the school transition. The decrease in social acceptance was congruent with expectations, and this may result from changes in adolescents’ social networks connected to school transition (Wigfield et al. 1991), as many adolescents face challenges finding a place in a new group of peers and teachers. An increase in behavioral conduct and job competence may stem from an increasing insight into self-responsibility regarding correctness of behavior and social expectancies with regard to “adult-like” behavior. In line with this interpretation, Crocetti et al. (2019), in their longitudinal study with middle adolescents, used items from the behavioral conduct subscale of the SPPA to measure self-perceived morality and also found that females’ morality increased over the 2-year period. However, one should note that, generally, at a group level, self-esteem seems rather continuous for most domains of self-esteem, as well as for global self-esteem, and no changes were observed following the school transition, indicating that this context may be not as challenging for the evaluative aspect of self-concept as one may have supposed based on earlier research (Cole et al. 2001; Harter et al. 1992; Simmons et al. 1987). Note that most of this research was conducted in the XX century, and thus the results of this study are important as the new insight in self-esteem development in XXI-century adolescents was provided.

It should be noted that the observed pattern of change in self-esteem was inter-individually differentiated, but, in general, the age group (early versus middle adolescence) was not responsible for this differentiation. In other words, although there generally were no differences between the age groups in self-esteem development based on the results of the conditional LGC, when age was taken as a covariate, likely in middle adolescence, changes in physical appearance and global self-esteem were more differentiated than in early adolescence. One may suggest that in comparison with previous research (Cole et al. 2001), the development of self-esteem as identity development (Klimstra et al. 2010) might be prolonged or delayed, and thus changes in the most subjectively important aspect of self-esteem in adolescence (Harter 2012b); i.e., global self-worth and physical appearance, are only observed in middle adolescents. However, this effect should also be further analyzed (probably with a larger sample) as at the whole group level, these two aspects of self-esteem did not change. Therefore, the hypothesis that a change or even a decrease in self-esteem in early adolescence may be more pronounced (Cole et al. 2001; Harter 2012b) was not confirmed. It may be supposed that, generally, school transition in adolescence may influence self-esteem in a similar way, independently of the level of education. On the other hand, taking into account that there are inter-individual differences in self-esteem development, the lack of age differences when the whole group was taken into account could suggest additional factors not taken into consideration in the present study that exist and influence different courses of self-esteem development. These additional factors may be related to peers’ social support (Hirsch and DuBois 1991), body image and relations with parents (Birkeland et al. 2012) or shifts in life events and family cohesion (Baldwin and Hoffmann 2002), as well as individual factors related to socio-economic status (SES), race or social class (Rhodes et al. 2004). Analyzing these factors definitely deserves further studies.

Although it cannot be completely confirmed that clear differences exist in the rate of change in self-esteem between early and middle adolescence, differences were found in the initial levels between age groups. Contrary to expectations, self-esteem was higher in the younger group (in the cases of scholastic competence, social acceptance, athletic competence, physical appearance and global self-esteem). No differences between age groups were found in close friendship, job competence, behavioral conduct or romantic appeal. The results highlight the complexity of self-esteem in the period of adolescence. In general, self-esteem was lower among middle adolescents in comparison to early adolescents; however, with regard to some domains, this rule was not observed. It may be speculated that the drop in self-esteem observed between childhood and adolescence (Baldwin and Hoffman 2002; Robins et al. 2002) is more prolonged, and the later observed increase in self-esteem (Orth et al. 2012) is more delayed. However, this is only an hypothesis, and the cross-sectional data do not allow for verification of this speculation. On the one hand, it may be concluded that the period of early adolescence does not seem as challenging for self-esteem as supposed (Harter 2012b), and young adolescents generally have a more positive view of their competences than their older counterparts. On the other hand, it must be emphasized that this conclusion should be considered cautiously as one of the studied age samples was gender-biased: in the middle adolescence group there were more girls than boys (107 versus 34); and thus the lower self-esteem in this middle adolescent group may be related to the gender or gender x age interaction, even though the meta-analyses on self-esteem trajectories indicated the same pattern for both males and females (Huang 2010; Trzesniewski et al. 2003). This suggests that gender is not a significant factor influencing the continuity or stability of self-esteem. It has been shown, however, that self-esteem tends to be lower in females than in males (Bleidorn et al. 2016; Kling et al. 1999; Moksnes and Espnes 2013; Quatman and Watson 2001), and this pattern may be more pronounced in middle adolescence (Kling et al. 1999). Therefore, the observed pattern of age differences in self-esteem may be intertwined with gender differences during this age period.

The pattern of results slightly changed at the individual level, when the rank-order stability of self-esteem was analyzed. The results of autoregressive analyses revealed that, for the younger group, global self-esteem just after the school transition explained only 14% of its variance 9 months later, even though at the group level, global self-esteem seemed stable. This is also true for behavioral conduct in this group (13% of variance at T2) and for romantic appeal (15% of variance at T3). For most domains of self-esteem, particularly in early adolescence, the level of self-esteem at T1 explained only a small part of its variance 9 months later. These results suggest that school transitions create a context for some kind of instability in self-esteem at the individual level. Afterward, during the more extended period of time, this stability seems to be restored, which seems congruent with the growing stability of individual differences reported in the literature (Trzesniewski et al. 2003).

### Limitations

This study’s conclusions have limitations worth noting. They are limited due to the specific context of school transitions in Poland, which may interact with developmental changes typical for the period of early and middle adolescence. Specifically, in early adolescence, there is a transition to junior high school, which comprises the last level of obligatory education in Poland. On the other hand, the transition to high school (in middle adolescence) is not obligatory, and it refers to about 50% of teenagers graduating from junior high schools. Additionally, the attrition rate was higher among middle adolescents, which may be related to a higher school absence rate in this age group (Motyka 2018).

One also can argue that short periods of time between measurement points may make observing developmental change in self-esteem difficult or may influence the observed developmental trends as usually the studied time periods have been much longer (e.g., Kuzucu et al. 2014). However, the short periods of time between measurement points enabled us to reveal short-term changes, observable particularly at the individual level (instability in self-esteem just after school transition), which may not be visible in a broader time perspective.

Moreover, the study was limited in terms of the participants and measures. The study mainly included participants from an urban area, so the sample was rather homogeneous. The sample was not equal regarding gender distribution as in the middle adolescent group, there were many more girls than boys (107 versus 34), and this factor could have influenced the obtained results, particularly regarding the level of self-esteem (Kling et al. 1999; Quatman and Watson 2001). It should also be highlighted that some of the presented results should be considered with caution as some of the analyzed models (for example, regarding behavioral conduct) did not reach acceptable fit indices, and the reliability coefficients of some of the SPPA scales were rather low. However, the reliability of the behavioral conduct subscale was also below 0.7 for the original version of the SPPA (Harter 2012a). Low alphas (below 0.6) for the romantic appeal scale in early adolescents might have been affected by the fact that they are not yet motivated to be interested in one romantic partner. On the other hand, middle adolescents might be not so focused on school achievements, and thus their answers on the scholastic competence subscale were not so consistent.

### Future directions and implications

This study’s results supported the validity of the adopted approach. The combination of cross-sectional and longitudinal designs and providing both group and individual analyses in one study allowed to adequately describe the complexity of the development of self-esteem in adolescence. Despite finding a general continuity in the development of self-esteem in the periods of early and middle adolescence, individual differences in the trajectories of development of self-esteem were also discovered. This likely means that, in some domains, the change is more complex. Thus, it may be suggested that future research is needed in the area of self-esteem in adolescence because the courses and the consequences of individual differences in the development of self-esteem should be analyzed in depth. One may speculate, for example, that the direction of change can vary: it can decrease in one subgroup, increase in another, and in the next maintain an equilibrium. This interpretation is also supported by the analyses of the changes at the individual level, suggesting some weak instability in self-esteem, particularly just after the school transition. Therefore, one should note that the general, group-pattern changes in self-esteem may not automatically refer to all individuals, as individual patterns of change may be much more differentiated. The present study only revealed the inter-individual differences in the course of the development of self-esteem; thus, additional analyses aimed at drawing a distinction between the groups with distinct courses of change in self-esteem are needed.

The need for more research seems particularly important in the context of practical implications. Different trajectories of self-esteem may lead to different developmental outcomes not only in adolescence (Zimmerman et al. 1997) but also throughout the course of one’s entire life (Steiger et al. 2014). The implications of such an observation for educational policy are very important, as people may require different supports at different life periods.

These implications are even more significant if one considers that self-esteem is a key determinant of well-being and success in life (Deci and Ryan 1995; James 1890/1952; Leary 1999). This research indicates that the development of self-esteem does not have a uniform trajectory. Firstly, self-esteem develops differently in particular domains and, secondly, it develops differently for different age groups during adolescence; both of these observations are important, as the precise description of these changes can provide guidance for those working with young people. For example, when assuming the key importance of self-esteem in the lives of young people, one should be aware that supporting people in early and late adolescence should be approached in different ways. According to Topolewska-Siedzik and Cieciuch (2018), these two periods in research and development are often treated together, without proper distinctions made between them. The specificity of each of these periods should be further tested, as results obtained confirm that younger and older adolescents develop differently and probably require different kinds of support during this process. This applies also to other developmental periods, as there is also a need to describe change and consistency in self-esteem in late adolescence and in emerging adulthood.

Last but not least, based on the present results, the theoretical implications of understanding the period of adolescence should be underscored, as adolescence may be viewed as a formative period of life (Blakemore 2018) and understanding how young people develop a sense of self (particularly, self-concept and self-esteem) may be seen as especially important. This is one of the most crucial challenges of this age group (James 1890/1952; Harter 2012b). Additionally, as the individual differences in the continuity and stability of different domains of self-esteem are observed, they may be related to changes in the importance of different domains of self-esteem in the course of adolescence generally (Harter 2012b). Definitely, the issue of the importance of different domains of self-esteem and their significance for global self-esteem also deserves future studies.

## Conclusion

Although the development of self-esteem in adolescence have been widely studied, previous results have been mixed and questions about patterns of change and consistency at both the mean (group) and the individual level have not been asked. In the present study, using multidimensional and multilevel designs, the complexity of the patterns of development of self-esteem during early and middle adolescence was described. First, in most domains of self-esteem and in global self-esteem over the period of a year-and-a-half, the continuity was observed, not change. Second, in most domains, a weak but quickly restored stability at the individual level was found. Third, also observed was higher self-esteem in early (not middle) adolescence and significant individual differences in the levels of global and domain-specific self-evaluations not explained by age. In general, at the beginning of the 21st century, school transitions turned out to be not as significant turning point as it had been previously thought.

## Footnotes

1 This attrition rate may be related to the fact that the second phase of the study was conducted in June, at the end of the school year, which is usually related to greater school absence.

2 The full questionnaire is available here: https://portfolio.du.edu/SusanHarter/page/44210
